# Structural identification of electron transfer dissociation products in mass spectrometry using infrared ion spectroscopy

**DOI:** 10.1038/ncomms11754

**Published:** 2016-06-09

**Authors:** Jonathan Martens, Josipa Grzetic, Giel Berden, Jos Oomens

**Affiliations:** 1 Radboud University, Institute for Molecules and Materials, FELIX Laboratory, Toernooiveld 7c, 6525ED Nijmegen, The Netherlands; 2 Van 't Hoff Institute for Molecular Sciences, University of Amsterdam, Science Park 908, 1098XH Amsterdam, The Netherlands

## Abstract

Tandem mass spectrometry occupies a principle place among modern analytical methods and drives many developments in the ‘omics' sciences. Electron attachment induced dissociation methods, as alternatives for collision-induced dissociation have profoundly influenced the field of proteomics, enabling among others the top-down sequencing of entire proteins and the analysis of post-translational modifications. The technique, however, produces more complex mass spectra and its radical-driven reaction mechanisms remain incompletely understood. Here we demonstrate the facile structural characterization of electron transfer dissociation generated peptide fragments by infrared ion spectroscopy using the tunable free-electron laser FELIX, aiding the elucidation of the underlying dissociation mechanisms. We apply this method to verify and revise previously proposed product ion structures for an often studied model tryptic peptide, [AlaAlaHisAlaArg+2H]^2+^. Comparing experiment with theory reveals that structures that would be assigned using only theoretical thermodynamic considerations often do not correspond to the experimentally sampled species.

## Abstract (web summary)

Mass spectrometry is a leading method used for sequencing peptides and proteins by fragmentation followed by analysis of the sequence fragments. Here, the authors use infrared spectroscopy to characterize the structures of peptide fragments formed during electron transfer dissociation.

## Graphical abstract

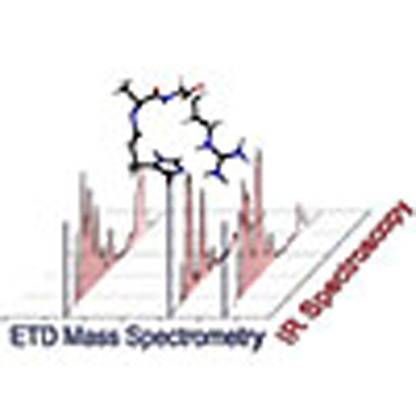

Mass spectrometry-based analysis of peptides and proteins in the bioanalytical and clinical sciences relies on the gas-phase dissociation of their molecular ions to give sequence fragments from which the original primary structure can be inferred. Sequencing by the increasingly popular electron-induced dissociation methods (ExD, such as electron capture and transfer dissociation, ECD and ETD) has recently seen rapid development and widespread application. ECD in Fourier transform ion cyclotron resonance (FT-ICR) mass spectrometry (MS)^1^,^2^,^3^ and more recently ETD in a much broader range of mass spectrometers^4^,^5^,^6^,^7^ constitute the two primary variations of this method. These methods have shown impressive improvements over collision-induced dissociation tandem MS, primarily in the sense that labile post-translational modifications are not detached during activation revealing their position along the backbone, and that sequence coverage is increased, making the sequencing of intact proteins in top-down strategies possible^8^,^9^,^10^,^12^.

Electron attachment to multiply protonated peptides or proteins leads to extensive backbone fragmentation, which most often takes the form of backbone N–C_α_ bond cleavages to provide the c- and z-type ion series^1^,^2^,^13^,^14^. The process of electron-induced N–C_α_ bond cleavage in peptide cation radicals has been the subject of many recent experimental and theoretical studies^13^,^15^,^16^,^17^,^18^,^19^,^20^. However, the mechanisms involved in electron attachment to peptide ions, the possible role of excited electronic states, and the structural rearrangements and fragmentation reactions that can follow, remain only partially understood. Furthermore, answers to questions regarding the ratios of fragment ions produced and their structures, especially the odd electron fragments, remain, at least partially, elusive. This is undoubtedly related to the uncertainty in the detailed nature of the (open-shell) dissociation products. For example, ExD product ions are known to undergo hydrogen atom migration reactions that increase/decrease the expected masses of the fragment ions, having direct practical implications for ExD-based sequencing applications^21^,^22^.

Several reaction mechanisms have been proposed for ExD of which the Cornell mechanism from the group of McLafferty^2^ and the more recent Utah-Washington (UW) mechanism from the groups of Tureček and Simons^19^,^23^,^24^,^25^ are best known. Frison *et al*.^26^ have discussed the different structures of c-type fragments that would result from different ECD mechanisms and were able to spectroscopically identify an amide c-type ion. While not being able to exclude the possibility that this structure results from a rearrangement after the ECD process, this assignment appears to support the UW mechanism, rather than mechanisms that would directly produce enol-imine c-type ions.

In ExD dissociation, the radical is typically on the z-type fragment, although H-atom migration reactions in the dissociating molecule may transfer the radical to the c-type fragment, resulting in changes in the *m*/*z* values of the fragments and complicating the interpretation of ExD MS/MS data. As a result, apparent unit mass shifts of backbone sequence ions in ExD spectra are thus not rare and result in an increased occurrence of ions with overlapping masses. As an illustration, for a peptide with the sequence AlaAlaHisAlaArg, hydrogen atom migration to the open-shell z_3_^·^^+^ fragment would give a closed-shell z_3_^+^ ion having the same chemical formula as the c_4_^+^ fragment and makes this mass peak unassignable for sequencing purposes.

Unfortunately, the detailed information about an ion's molecular structure is scarce and often undecipherable from MS/MS data alone. Infrared spectroscopy allows the structural characterization of both the trapped precursor and fragment ions. Infrared ion spectroscopy (IRIS) has been used extensively to determine the gas-phase structures of molecular ions in MS, and specifically in the effort to elucidate peptide fragmentation mechanisms involved in collisional dissociation^27^,^28^,^29^,^30^,^31^,^32^,^33^,^34^. Tunable infrared free-electron lasers (FELs), such as FELIX at our institute^35^, have played an important role in the recent development of IRIS^36^, and are especially useful to obtain fingerprint infrared spectra of ionic species on commercial MS platforms^37^. However, to date, only a single example of infrared spectroscopy on ECD-generated fragments has been reported, in which a small closed-shell c-type ion was examined^26^.

Structural characterization of peptidic ExD product ions is something that has been highly sought after for a number of years using a variety of methods, including collisional activation^38^,^39^, ion mobility methods^40^ and more recently ultraviolet photo-dissociation studies^41^,^42^,^43^. However, in comparison with infrared spectroscopy these methods provide limited information, regarding the structure and conformation of gas-phase ions. Structural characterization of the dissociation products by infrared spectroscopy and quantum chemistry provides a stringent test to ascertain and confirm uncertain aspects of the fragmentation mechanisms and allows for additional information to be extracted from MS/MS data. Here we present the first direct structural characterization of ETD-generated fragments using IRIS and demonstrate the strengths of this technique for addressing the questions surrounding gas-phase peptide radicals.

## Results

### ETD MS/MS of [AAHAR+2H^+^]^2+^

**Figure 1:**
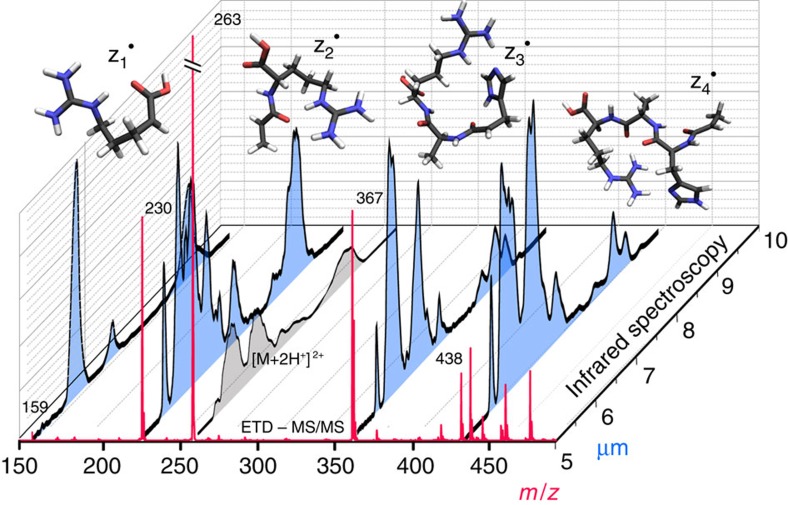
The ETD MS/MS spectrum of [AAHAR+2H]^2+^ with the corresponding infrared spectra. The infrared spectra of the ETD-generated fragments are shown in black/blue and that of the precursor peptide in black/grey. Supplementary Fig. 1 contains the comprehensive ETD MS/MS results.

**Figure 2:**
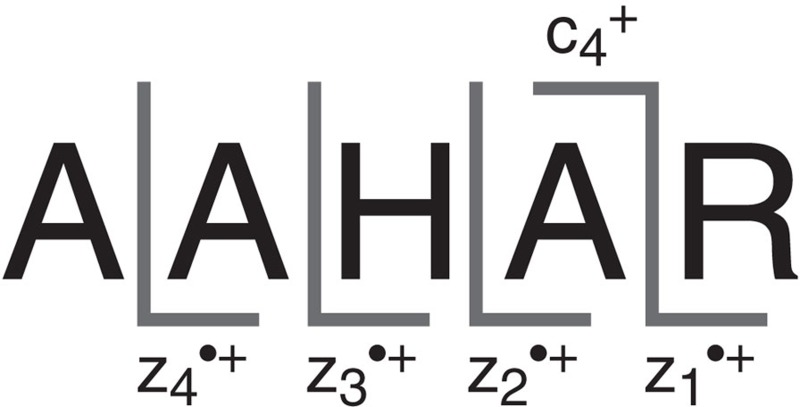
Dissociation scheme and notation used for product ions. c- and z-type peptide fragments typically result from ETD MS/MS. Here we label only the discussed sequence ions from ETD of [AAHAR+2H]^2+^. Note that fragments carrying a ‘·' symbol are open-shell radicals and those without are closed shell.

The ETD MS/MS spectrum of the [AAHAR+2H]^2+^ (263 *m*/*z*) ion is presented in red in Fig. 1. We have characterized each of the z^·^-type fragments (depicted in the peptide sequence shown in Fig. 2) from the ETD MS/MS spectrum using infrared spectroscopy, providing a comprehensive identification of their structures and conformations. This detailed characterization forms a basis for analysing the reactions, leading to their formation.

Tryptic peptides of the AAXAR type and their ETD MS/MS behaviour have been extensively studied previously^39^,^42^,^44^,^45^,^46^,^47^. Here, consistent with previously reported results, we observe that fragment ion intensity is approximately split over the charge reduced ion (ETnoD) and z^·^-type sequence ions (Supplementary Fig. 1). For AAHAR, having arginine in the C-terminal position, it is not surprising that C-terminal z^·^-type fragments primarily retain the proton after dissociation and that they are the principle fragments in the ETD MS/MS spectrum. The electron attachment process is no doubt affected by the protonation sites and conformation of the parent peptide. In its doubly protonated state, this peptide has been shown to protonate on the imidazole group of the histidine side chain and the guanidine group of the arginine side chain^45^, and our spectroscopic data and calculations confirm this.

### Structure of [AAHAR+2H^+^]^2+^ precursor peptide at *m*/*z* 263

**Figure 3:**
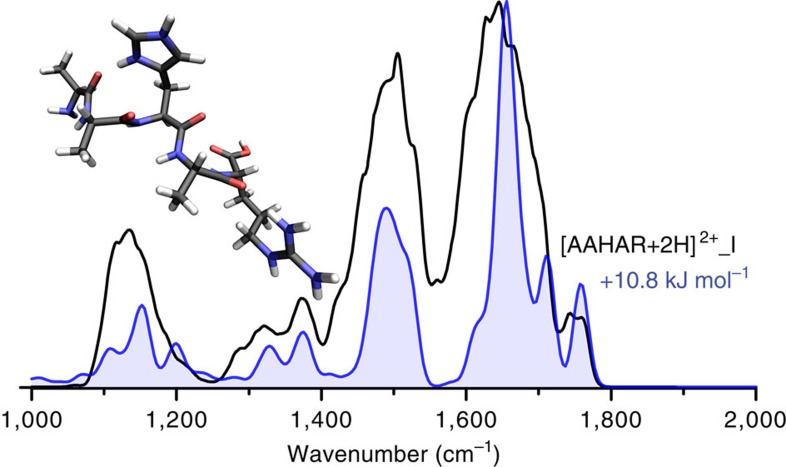
The infrared spectrum of [AAHAR+2H]^2+^ 263 *m*/*z*. The experimental spectrum is presented in black and the spectrum of the assigned calculated structure from this study is shown in blue along with the structure and relative free energy at 298 K.

Figure 3 presents the infrared spectrum obtained for the doubly protonated peptide [AAHAR+2H]^2+^ at *m*/*z* 263. Comparison with the calculated spectrum (blue) allows assignment of the protonation sites (His and Arg sidechains) and the conformation to be made. The band just <1,800 cm^−1^ is consistent with the free (or weakly H-bonded) carboxyl group. Both charged sites are hydrogen bound to backbone carbonyl groups. In terms of hydrogen bonding, involving the charged sites and the C and N termini, this assignment confirms a previously proposed structure^44^, although it has an overall more extended conformation, easily distinguished by comparison with the infrared spectra (Supplementary Fig. 2).

For singly charged fragments retaining the His residue, the imidazole ring can tautomerize (having the H on either the N1 or N3 position) and we address this issue for the z_3_^·^^+^ and z_4_^·^^+^ fragments. Note that all fragments carrying a ‘·' symbol are open-shell radicals and those without are closed shell, where H's and electrons are implicit. Backbone sequence fragments studied here with the structural identification based on our spectroscopic results, as detailed in the following are summarized in Table 1.

### z_1_^·^^+^ fragment structure

**Figure 4:**
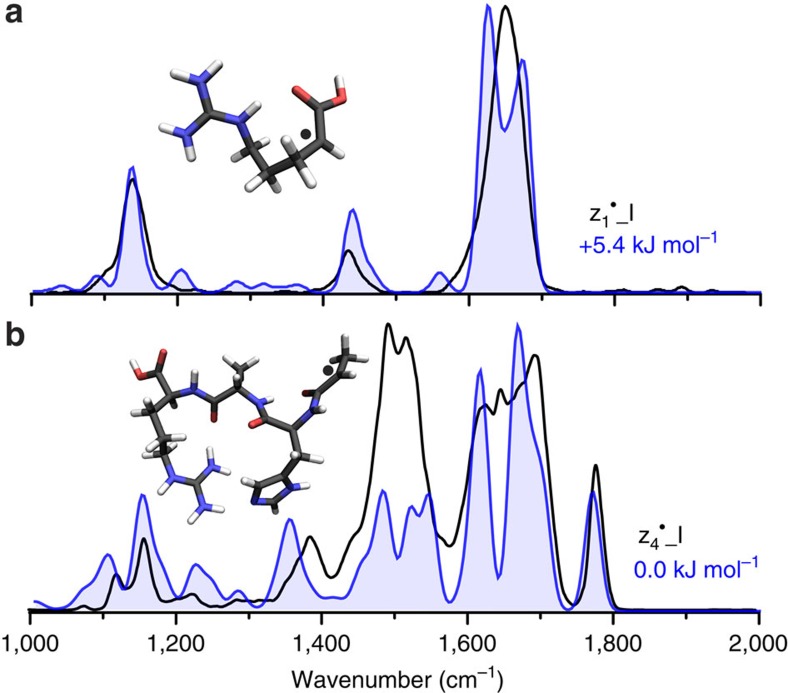
The infrared spectra of the z_1_^·^^+^ and z_4_^·^^+^ fragments from ETD of [AAHAR+2H]^2+^. The experimental spectra are presented in black in both cases and assigned calculated structures are shown in blue for (**a**) the z_1_^·^^+^ fragment and (**b**) the z_4_^·^^+^ fragment. The associated relative free energies (298 K) and structures are inlayed with the radical sites labelled by ‘·'.

The z_1_^·^^*+*^ fragment is the smallest C-terminal fragment obtained upon ETD of the precursor peptide. Calculated structure z_1_^·^_I was identified to match most closely with experiment, as demonstrated in the top panel of Fig. 4. This structure is the lowest-energy calculated structure from our selection of ∼30 structures from the molecular dynamics (MD) procedure. In structure z_1_^·^_I, the radical is located at the α-carbon adjacent to the C terminus and the carboxyl C=O stretch is found at 1,635 cm^−1^. On the basis of this structure, radicals at other positions along the carbon chain of this fragment were defined and optimized, most of which were higher in energy. H-atom migration to the δ-carbon of the Arg side chain gave a structure 5.4 kJ mol^−1^ more stable; however, a calculated barrier of 115.2 kJ mol^−1^ (Supplementary Fig. 3) must be overcome to reach it and it is not consistent with the experimental infrared spectrum. Barriers of >100 kJ mol^−1^ for H-atom migrations have been reported for different ETD fragments^39^,^48^,^49^. With the radical in the α-position, conjugation with the carbonyl occurs giving partial double-bond character to the CC-bond, while lowering the bond order of the carbonyl, causing a significant red shift of its stretching mode relative to a carbonyl stretch >1,700 cm^−1^ in the β-, γ- and δ-radicals (Supplementary Fig. 4). This is a clear demonstration that the position of the radical can strongly influence the vibrational spectrum, in this case the C=O stretch, highlighting the value of infrared spectroscopy for characterizing open-shell peptide fragments.

### z_2_^·^^+^ fragment structure

**Figure 5:**
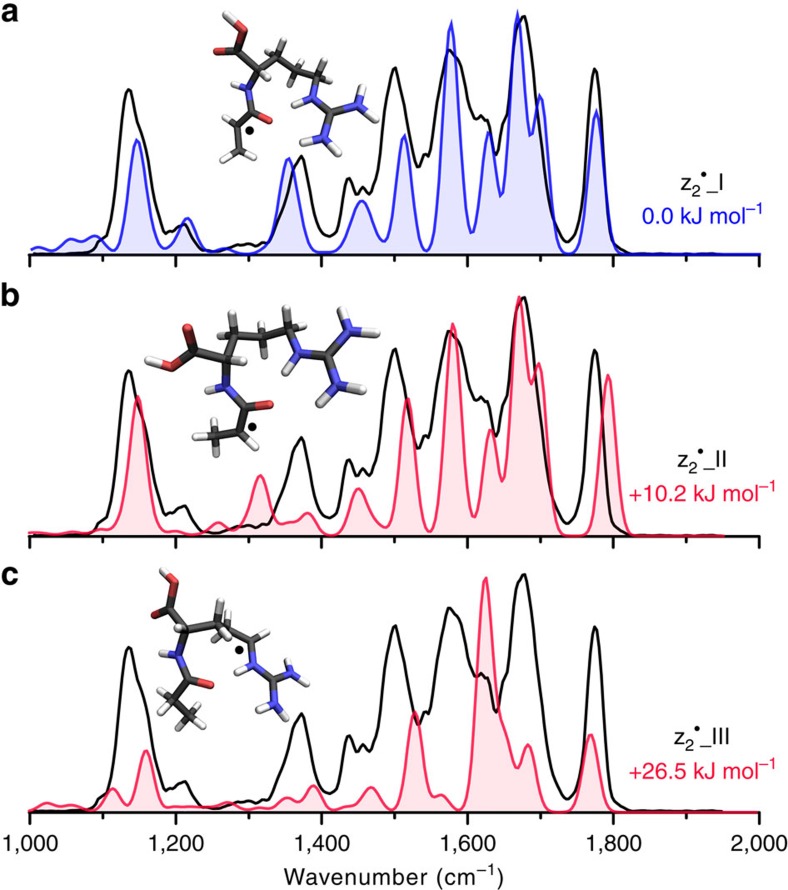
The infrared spectrum of the z_2_^·^^+^ fragment from ETD of [AAHAR+2H]^2+^. The experimental spectrum is presented in black and is compared with computed spectra for different low-energy structures. (**a**) The calculated spectrum for the assigned structure is shown in blue. (**b**,**c**) The calculated spectra for structures disregarded on the basis of spectral mismatch are shown in red. Calculated structures and relative free energies (298 K) are inlayed for each plot.

Figure 5 and Supplementary Fig. 5 present infrared spectra for the z_2_^·^^+^ fragment from ETD of [AAHAR+2H^+^]^2+^. The calculated spectrum of z_2_^·^_I, presented in the top panel, matches the experimental spectrum well. This structure is the lowest-energy calculation obtained from our computational procedure. A previously proposed structure^39^, labelled here as z_2_^·^_II and presented in the centre panel, closely resembles z_2_^·^_I. However, the alternate hydrogen bonding orientation of the C terminus is distinguished by comparison with experimental and calculated infrared spectra in the 1,300–1,400 cm^−1^ region and the carbonyl stretching region just <1,800 cm^−1^. This refinement of the conformation reduces the relative energy by 10.2 kJ mol^−1^. The bottom panel in the figure shows a comparison with the infrared spectrum calculated for the product of hydrogen migration from the α-carbon of the Ala residue to the δ-carbon of the Arg residue, a species 26.5 kJ mol^−1^ higher in energy and readily distinguishable spectroscopically.

### z_3_^·^^+^ fragment structure

**Figure 6:**
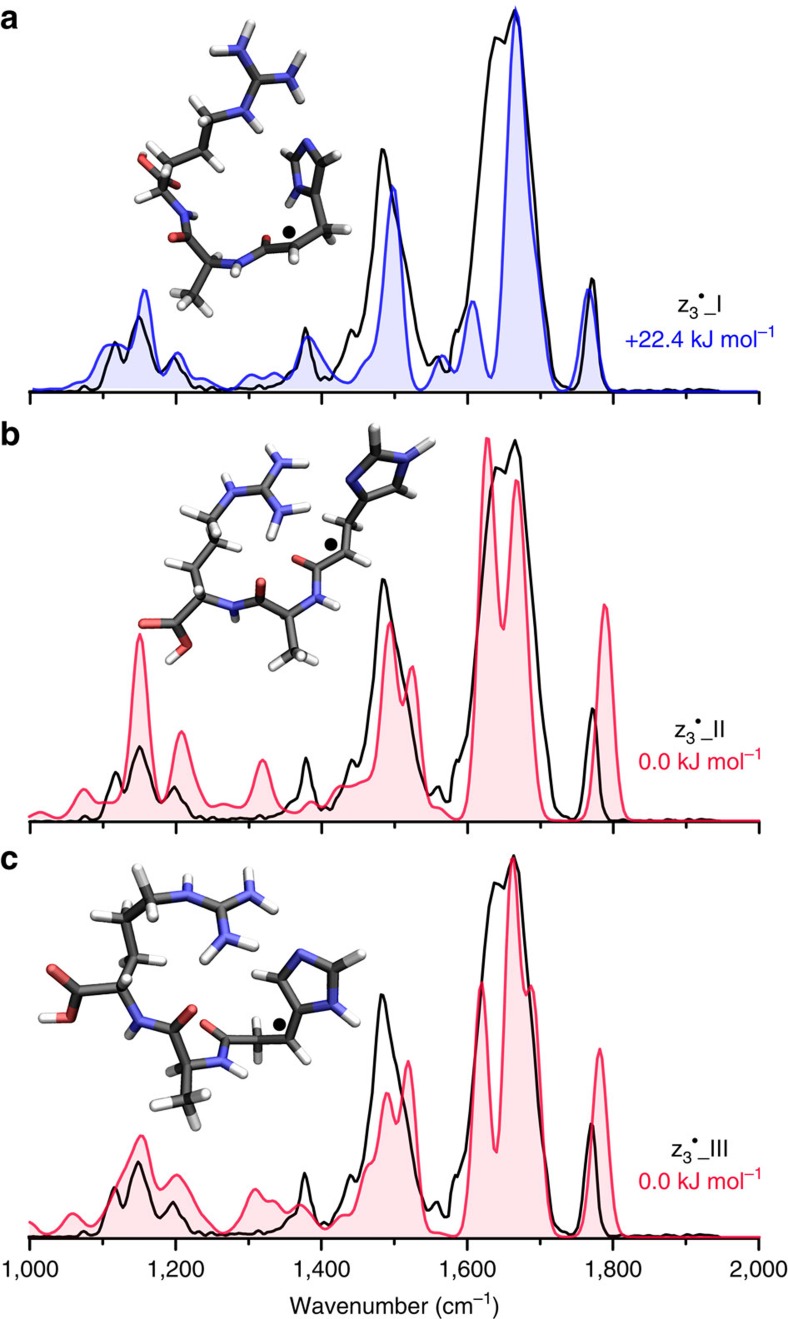
The infrared spectrum of the z_3_^·^^+^ fragment from ETD of [AAHAR+2H]^2+^. The experimental spectrum is presented in black and is compared with computed spectra for different low-energy structures. (**a**) The calculated spectrum for the assigned structure is shown in blue. (**b**,**c**) The calculated spectra for structures disregarded on the basis of spectral mismatch are shown in red. Calculated structures and relative free energies (298 K) are inlayed for each plot.

Figure 6 presents the infrared spectrum for the z_3_^·^^+^ fragment from ETD of [AAHAR+2H^+^]^2+^ with the spectrum of the assigned calculated structure z_3_^·^_I in blue and spectra of unassigned alternative structures in red. This assignment was made after considering different conformers, imidazole tautomers and products, resulting from hydrogen atom migration interconverting between the α-radical and the β-radical on the His side chain. A low-energy His N1 tautomer (z_3_^·^_II) was identified to be 22.4 kJ mol^−1^ lower in energy than z_3_^·^_I, a His N3 tautomer; however, the calculated spectrum of this species does not match as well to the experiment, especially in the 1,200–1,400 cm^−1^ region (and the 3400–3600, cm−1 region displayed in Supplementary Fig. 6). Furthermore, a β-radical structure (z_3_^·^_III) was found to be 22.4 kJ mol^−1^ more stable, but we do not assign this species on the basis of its spectral mismatch (Fig. 6 and Supplementary Fig. 6). The majority of stable structures we identified for each structure/tautomer of the z_3_^·^^+^ ion features a stabilizing hydrogen bonding interaction between the imidazole side chain of His and the guanidinium side chain of Arg, leaving little flexibility for the orientation of the carboxyl group and giving a free C–OH group and a C=O weakly hydrogen bonded to the adjacent amide N–H. Being very sensitive to local environment, the position of the carboxyl C=O stretch just <1,800 cm^−1^ can be used as a diagnostic signature. Structures z_3_^·^_II and z_3_^·^_III feature hydrogen bonds between the imidazole nitrogen and hydrogens of the two primary nitrogens of the guanidinium group, while in z_3_^·^_I the hydrogen bond of the imidazole nitrogen is shared between the hydrogens of the secondary nitrogen and one primary nitrogen.

### z_4_^·^^+^ fragment structure

The bottom panel of Fig. 4 presents the infrared spectrum for the z_4_^·^^+^ fragment from ETD of [AAHAR+2H^+^]^2+^ and the assigned calculated structure (blue). This is the overall lowest-energy structure obtained after an extensive MD-based search over different conformers for various tautomers and structures obtained by hydrogen migration from the α-radical to the His β-radical (see comparison of z_4_^·^_IV in Supplementary Fig. 7). Similar to z_3_^·^_I, the assigned structure, z_4_^·^_I, has a hydrogen bond between the imidazole side chain and the guanidinium group of the Arg residue. Structure z_4_^·^_I offers a refinement over a previously proposed structure^39^ (here, re-optimized at the currently applied level of theory), giving both a better spectral match (see z_4_^·^_II in Supplementary Fig. 7) and being ∼60 kJ mol^−1^ lower in energy.

### Fragment ion at *m*/*z* 368 can be z_3_^+^ or c_4_^+^

While inter- and intramolecular (between c and z fragment pairs) hydrogen migration reactions are commonly observed in ETD MS/MS, their behaviour is still relatively weakly understood. For [AAHAR+2H^+^]^2+^, only for the z_3_^·^^+^ cation do we observe an appreciable extent of such a reaction, where we see both the open-shell z_3_^·^^+^ fragment (*m*/*z* 367) and a closed-shell z_3_^+^ fragment (*m*/*z* 368). Highlighting the complications that can arise from hydrogen atom migrations in ETD, the c_4_^+^ fragment (C_15_N_7_H_25_O_4_) has the same chemical formula and overlaps the closed-shell z_3_^+^ fragment also at *m*/*z* 368. Identification and consideration of the hydrogen migration products (loss/gain) are important for assigning fragment ions and correct sequencing^38^.

**Figure 7:**
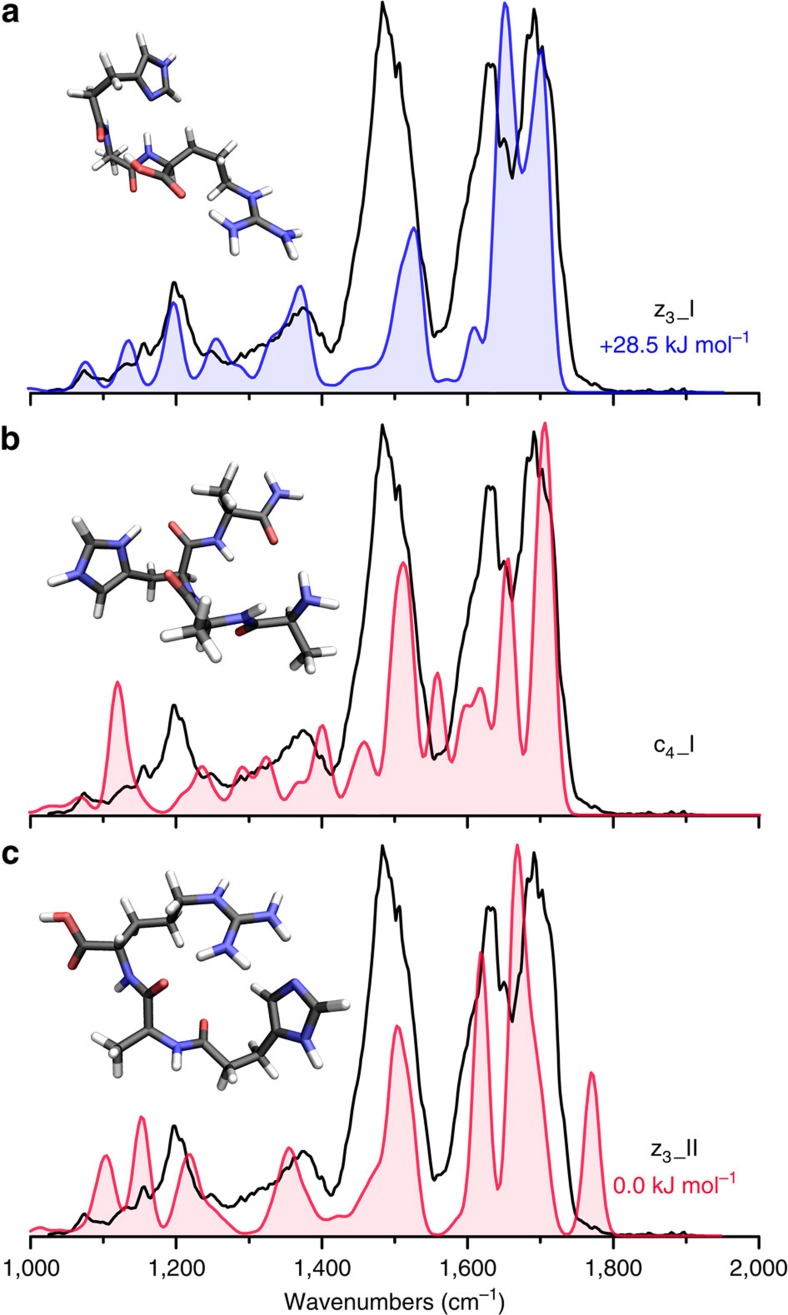
The infrared spectrum of the *m*/*z* 368 fragment from ETD of [AAHAR+2H]^2+^. The experimental spectrum is presented in black and is compared with computed spectra for different low-energy structures. (**a**) The spectrum of the assigned calculated closed-shell z_3_^+^ structure is shown in blue with the structure and relative free energy (298 K) inlayed. (**b**) c_4__I is a low-energy c_4_^+^ conformation and its calculated infrared spectrum is presented in red. (**c**) z_3__II is the lowest energy calculated z_3_^+^ structure identified in this work.

In Fig. 7, we identify the *m*/*z* 368 fragment ion as the z_3_^+^ species (z_3__I) based on infrared spectral matching. This structure features an alternative hydrogen bonding arrangement in comparison with the open-shell z_3_^·^^+^ and z_4_^·^^+^ fragments described above, most significantly affecting the C terminus (trans configuration) and red shifting the carboxyl C=O stretch for z_3__I away from the position just <1,800 cm^−1^ for the open-shell z_3_^·^^+^ and z_4_^·^^+^ fragments. A closed-shell equivalent of the geometry of the open-shell z_3_^·^_I structure is defined as z_3__II and is 28.5 kJ mol^−1^ lower in energy than z_3__I. Calculated spectra for structure z_3__II and c_4__I, a low-energy c_4_^+^ conformation, are presented in the bottom two panels of Fig. 7 and support the assignment of z_3__I.

## Discussion

These results demonstrate the first use of IRIS to characterize the structures of ETD-generated peptide fragments. Using a model tryptic pentapeptide, precursor ion conformation has been related to the observed ETD fragmentation pattern, and the structures and conformations of the various fragment ions. We show that it is possible to distinguish both conformational details and different radical species, when this approach is combined with routine computational modelling.

We conclude that if structural assignments were made only on the basis of theoretical (thermodynamic) considerations, these assignments would in many cases not match the species observed in experiment—highlighting the potential for IRIS to diagnostically identify gas-phase organic radicals and, more specifically, the mechanisms associated with peptide fragmentation in electron attachment methods. Our results suggest that hydrogen atom transfer necessary for radical migration often does not occur after ETD (without additional activation), leading to the frequent observation of non-equilibrium product ions. Understanding intermolecular hydrogen atom migration is also of practical importance, as it causes shifts in ETD fragment masses and makes sequence ion assignments more complicated.

## Methods

### Ion spectroscopy in a modified ion trap mass spectrometer

The experiment is based on a commercial quadrupole ion trap mass spectrometer (Bruker, AmaZon Speed ETD) coupled to the infrared beam line of the FELIX FEL. [M+2H]^2+^ peptide ions are generated by electrospray ionization. AAHAR (GeneCust (Luxemburg), 95% purity) solutions of 10^−5^–10^−6^ mol l^−1^ (in 50:50 acetonitrile:water, ∼1% formic acid) are introduced at 120 μl h^−1^ flow rates and desolvated by a pressurized nebulizing gas (N_2_). The key hardware modifications to the instrument providing optical access to the ion population in the trap were the introduction of a new ring electrode having 3 mm holes at its top and bottom, the installation of mirrors below the trap to direct the beam back out of the instrument and optical windows in the vacuum housing. In ETD experiments, ions were accumulated for 0.1–15 ms in the trap, mass isolated and then reacted with fluoranthene radical anions for ∼250 ms. A fragment ion of interest was mass isolated in a subsequent MS/MS stage and irradiated by the tunable infrared beam from the FEL. In the experiments reported here, the FELIX FEL was set to produce infrared radiation in the form of 5–10 μs macropulses at 5 or 10 Hz and of  30–60 mJ (bandwidth ∼0.4% of the centre frequency). Resonant absorption of infrared radiation leads to an increase in the internal energy of the molecule aided by intramolecular vibrational redistribution of the absorbed energy. When a sufficient number of photons is absorbed (here, typically in a single macropulse), unimolecular dissociation occurs and produces frequency-dependent fragment ion intensities in the mass spectrometer (Supplementary Note 1). Relating the parent and fragment ion intensities in the observed mass spectral data (yield=ΣI(fragment ions)/ΣI(parent+fragment ions)) generates an infrared vibrational spectrum. The yield at each infrared point is obtained from averaged mass spectra and is linearly corrected for laser power; the frequency is calibrated using a grating spectrometer.

### Computational chemistry

We have employed a molecular mechanics (MM)/MD approach using AMBER 12 (refs 50, 51). Molecular structures manually defined based on chemical intuition where first optimized for each ion at the B3LYP/6–31++G(d,p) level in Gaussian09 (ref. 52). Restrained electrostatic potential (RESP) charges from these initial results were used for parameterization of the nonstandard peptide ions in the antechamber program. After minimization within AMBER, a simulated annealing procedure up to 1,000 K was used with a 1 fs step size. Five hundred structures were obtained as snapshots throughout the procedure and after MM minimization were grouped based on structural similarity using *prtraj* in AMBER. Of these, 30–50 unique structures were then each optimized at the B3LYP/6–31++G(d,p) level^51^,^53^,^54^ and vibrational spectra were calculated within the harmonic oscillator model (vibrational frequencies were scaled by 0.975). This computational approach was applied to all structural isomers considered for each ion, except for the small z_1_^·^^+^ ion, where the MM/MD conformational search was only applied once using the alpha-radical species. Calculated line spectra were broadened using a Gaussian function with a full-width at half-maximum of 25 cm^−1^ to facilitate comparison with experiment. Additional calculations using the LC-BLYP and M06 functionals for a selection of z_1_^·^^*+*^ and z_2_^·^^*+*^ structures, and ab initio MP2 calculations for the z_1_^·^^*+*^ structures were performed to verify the validity of the choice of functional^49^,^55^ and these results are summarized in Supplementary Table 1 and Supplementary Fig. 8. In general, vibrational frequencies were found to be best modelled at the B3LYP/6–31++G(d,p) level and calculated free energies are mostly consistent between these levels of theory. Supplementary Data 1, 2, 3, 4 contain optimized geometries of the assigned z-type fragments.

### Data availability

The data that support the findings of this study are available from the corresponding author upon request.

## 

## Additional information

**How to cite this article:** Martens, J. *et al*. Structural identification of electron transfer dissociation products in mass spectrometry using infrared ion spectroscopy. *Nat. Commun.* 7:11754 doi: 10.1038/ncomms11754 (2016).

## Supplementary Material

Supplementary Information Supplementary Figures 1-8, Supplementary Table 1, Supplementary Note 1 and Supplementary References

Supplementary Data 1 Optimized coordinates for assigned structure z1

Supplementary Data 2 Optimized coordinates for assigned structure z2

Supplementary Data 3 Optimized coordinates for assigned structure z3

Supplementary Data 4 Optimized coordinates for assigned structure z4

## Figures and Tables

**Table 1:** Summary of structural properties and relative free energies for selected calculated structures.

	*m*/*z*	His tautomer	Radical	Rel. Δ*G* (kJ mol^−1^)
[AAHAR+2H^+^]^2+^	263			
I		—	—	+10.8*
II		—	—	0.0
z_1_^·^^+^	159			
I		—	α	+5.4*
II		—	δ (Arg)	0.0
III		—	β (Arg)	+10.1
IV		—	γ (Arg)	+14.7
z_2_^·^^+^	230			
I		—	α	0.0*
II		—	α	+10.2
III		—	δ (Arg)	+26.5
z_3_^·^^+^	367			
I		N3	α	+22.4*
II		N1	α	0.0
III		N3	β (His)	0.0
z_4_^·^^+^	438			
I		N3	α	0.0*
II		N3	α	+62.5
III		N3	—	+54.4
IV		N3	β (His)	+5.7
z_3_^+^	368			
I		N1	—	+28.5*
c_4__I		—	—	—
II		N3	—	0.0

Rel., relative.

An asterisk (*) indicates the structure assigned spectroscopically.
