# Supplementary material for: Structural identification of electron transfer dissociation products in mass spectrometry using infrared ion spectroscopy
Source: Nat Commun. 2016 Jun 9;7:11754. doi: 10.1038/ncomms11754 (PMC4906228; doi:10.1038/ncomms11754)
Supplement: Supplementary Information — Supplementary Figures 1-8, Supplementary Table 1, Supplementary Note 1 and Supplementary References [file ncomms11754-s1.pdf]

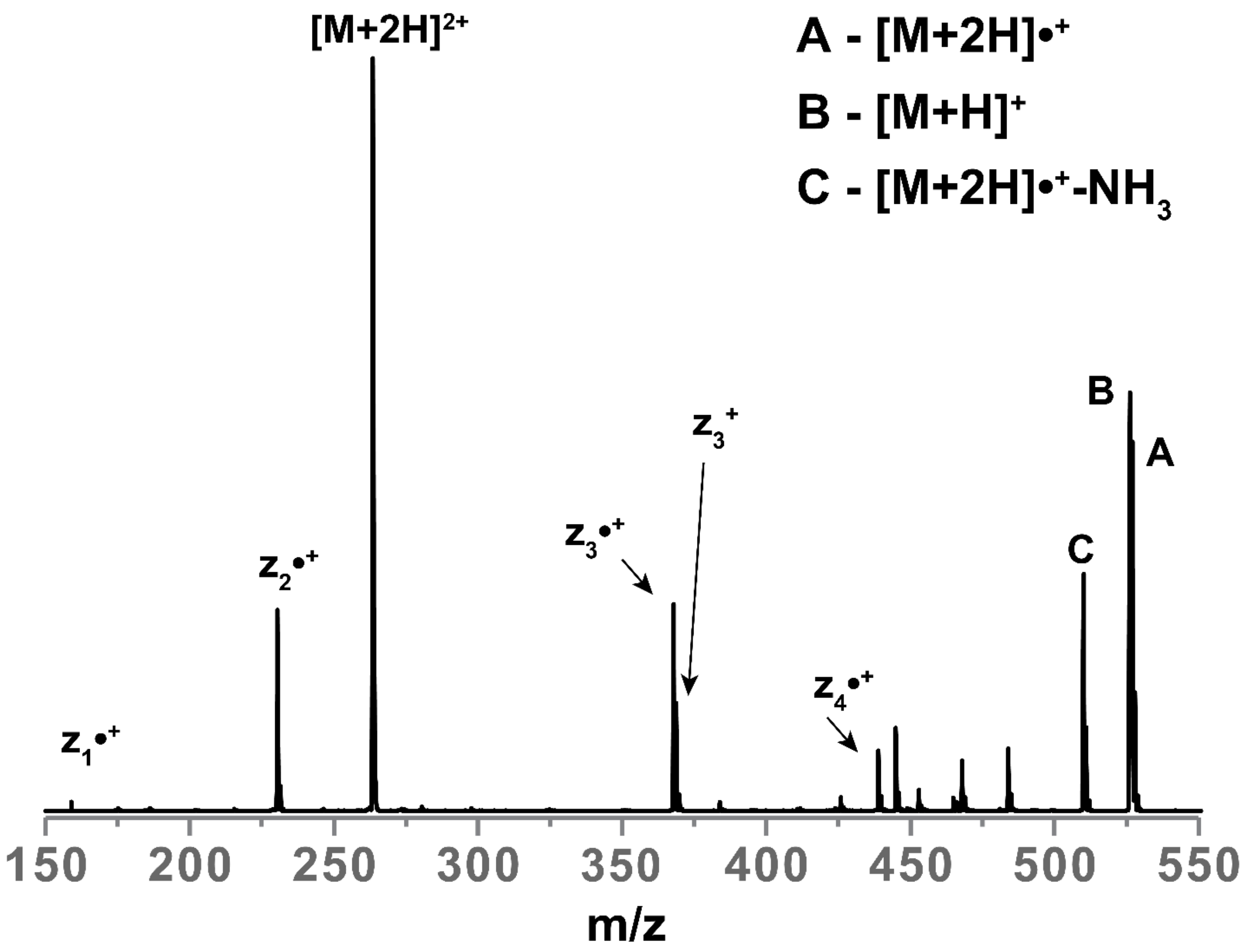


**Supplementary Figure 1 | The ETD MS/MS spectrum of $[AAHAR+2H]^{2+}$.**

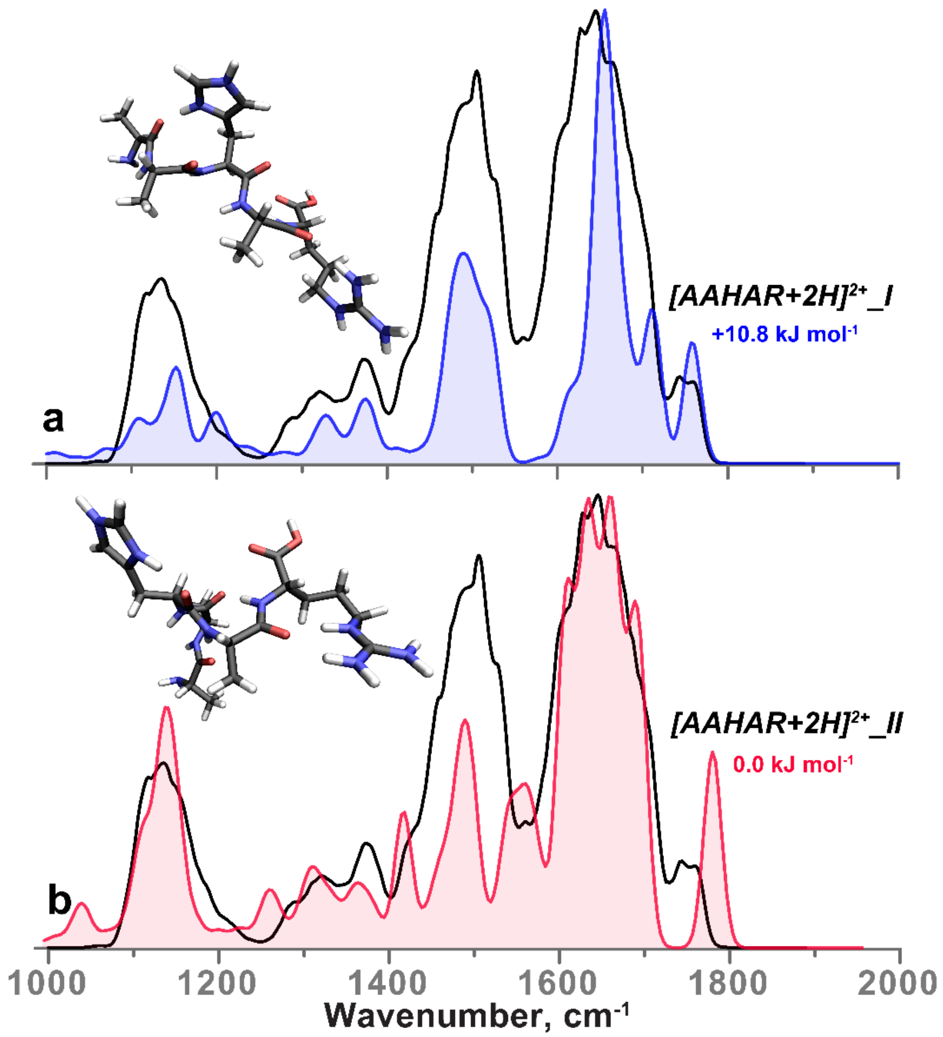


**Supplementary Figure 2 | The infrared spectrum of $[AAHAR+2H]^{2+}$.** The experimental spectrum is presented in black and calculated structures and relative free energies (298 K) are inlayed for each plot in both panels. **(a)** The assigned calculated structure is shown in blue. **(b)** $[AAHAR+2H]^{2+}$_II is based on a previously proposed structure[1] reoptimized at the level of theory employed in the present study.

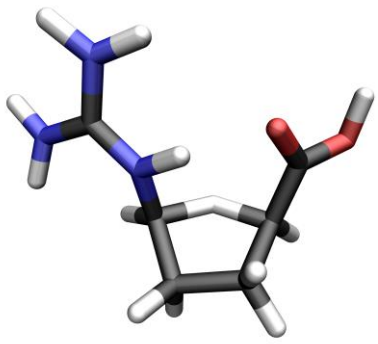

**Supplementary Figure 3 | Structure of the transition state for H-atom migration in the $z_1\bullet^+$ fragment produced by ETD of $[AAHAR+2H]^{2+}$.** In this structure, migration is taking place from the α-carbon to the δ-carbon of the Arg side chain.

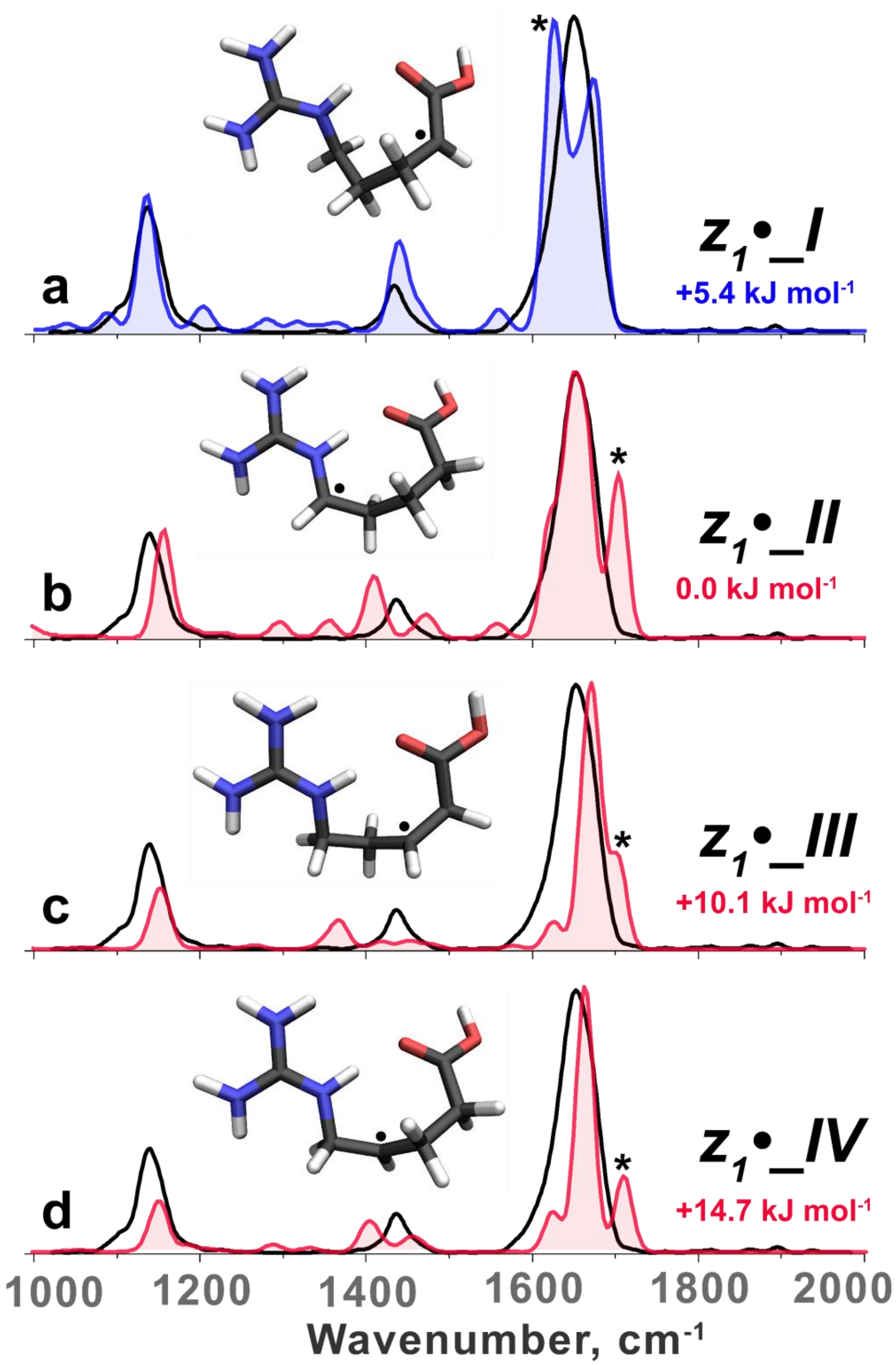


**Supplementary Figure 4 | The infrared spectrum of the $z_1$•$^+$ fragment from ETD of [AAHAR+2H]$^{2+}$. (a)** $z_1$•_I is the assigned calculated structure and is shown in blue. **(b)** $z_1$•_II, **(c)** $z_1$•_III and **(d)** $z_1$•_IV are structures that differ by H-atom migration along the Arg side chain and are presented in red in each panel. "*" labels indicate the bands having predominantly carbonyl stretching character. Calculated structures and relative free energies (298 K) are inlayed for each plot.

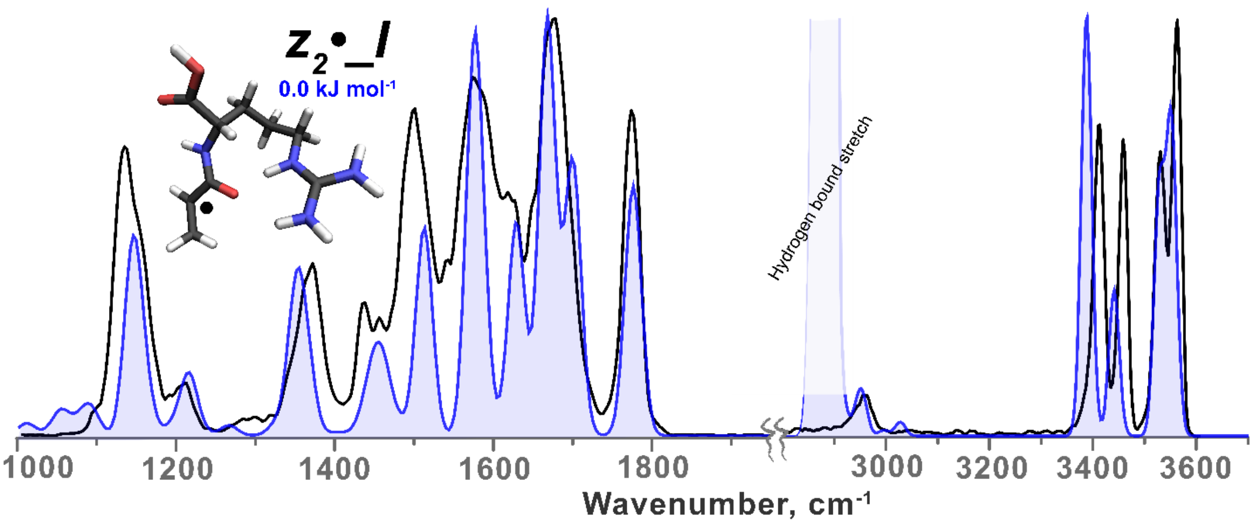


**Supplementary Figure 5 | The infrared spectrum of the $z_2$•$^+$ fragment from ETD of [AAHAR+2H]$^{2+}$ including the 3 micron spectral region**. The experimental spectrum is presented in black and the calculated spectrum for the assigned $z_2$•_I structure is in blue. IR measurements in the 3 micron region were obtained using an optical parametric oscillator/amplifier source (OPO/OPA) and support the structural assignment. Calculated frequencies in this region are scaled by 0.95. Stretching vibrations of hydrogen bonded protons are known to be poorly modelled within the harmonic oscillator approximation. In experiments, these bands are expected to be heavily broadened and red-shifted and often unobserved[2].

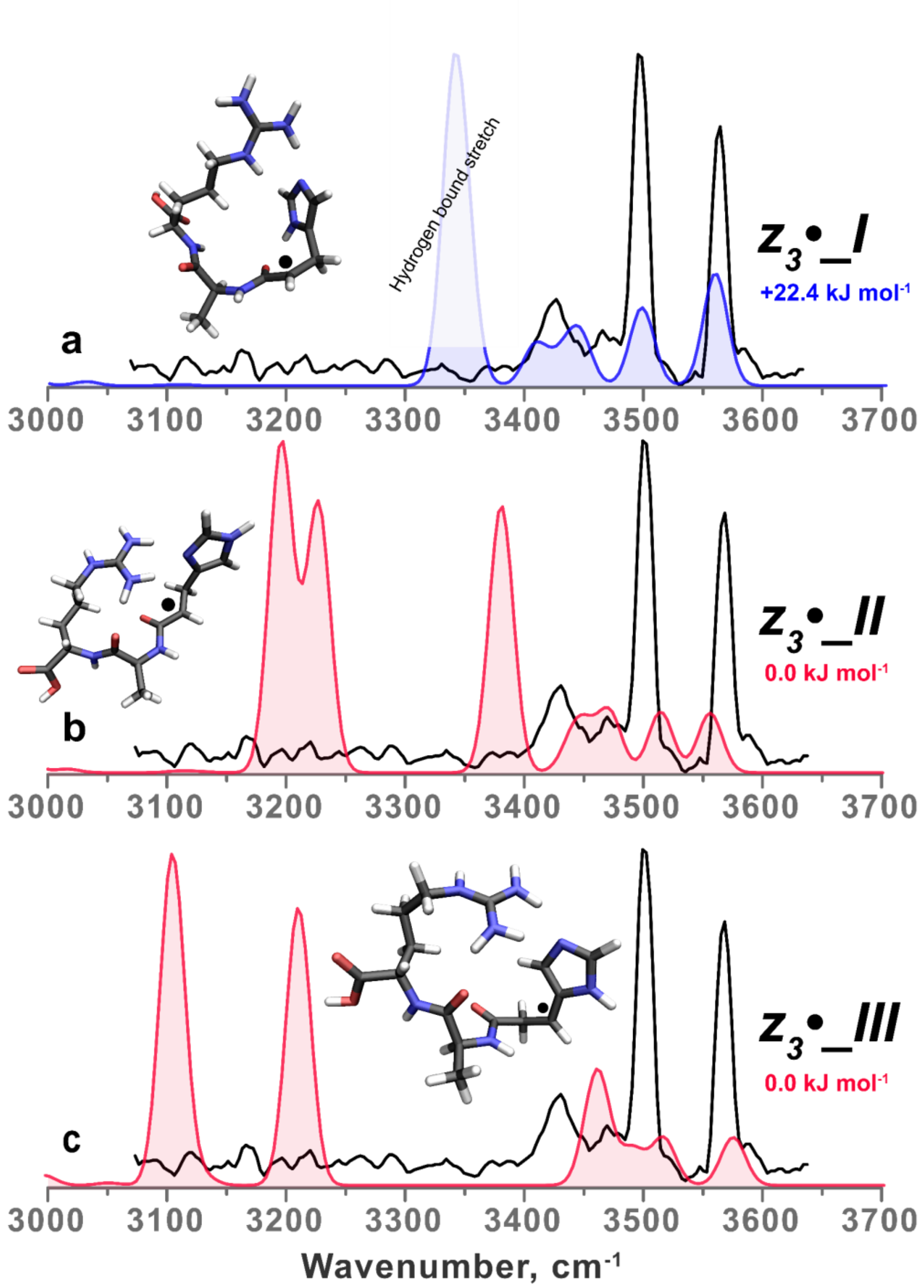


**Supplementary Figure 6 | The infrared spectrum of the $z_3$•$^+$ fragment from ETD of [AAHAR+2H]$^{2+}$in the 3.0 micron spectral region. (a)** The calculated spectrum for the assigned structure is in blue. **(b)** and **(c)** present calculated spectra for structures disregarded on the basis of spectral mismatch. Calculated structures and relative free energies (298 K) are inlayed for each plot. IR measurements in the 3 micron region (OPO) support the structural assignment made from the fingerprint region (FELIX) spectra. Calculated frequencies in this region are scaled by 0.95. Stretching vibrations of hydrogen bonded protons are known to be poorly modelled within the harmonic oscillator approximation.[2]

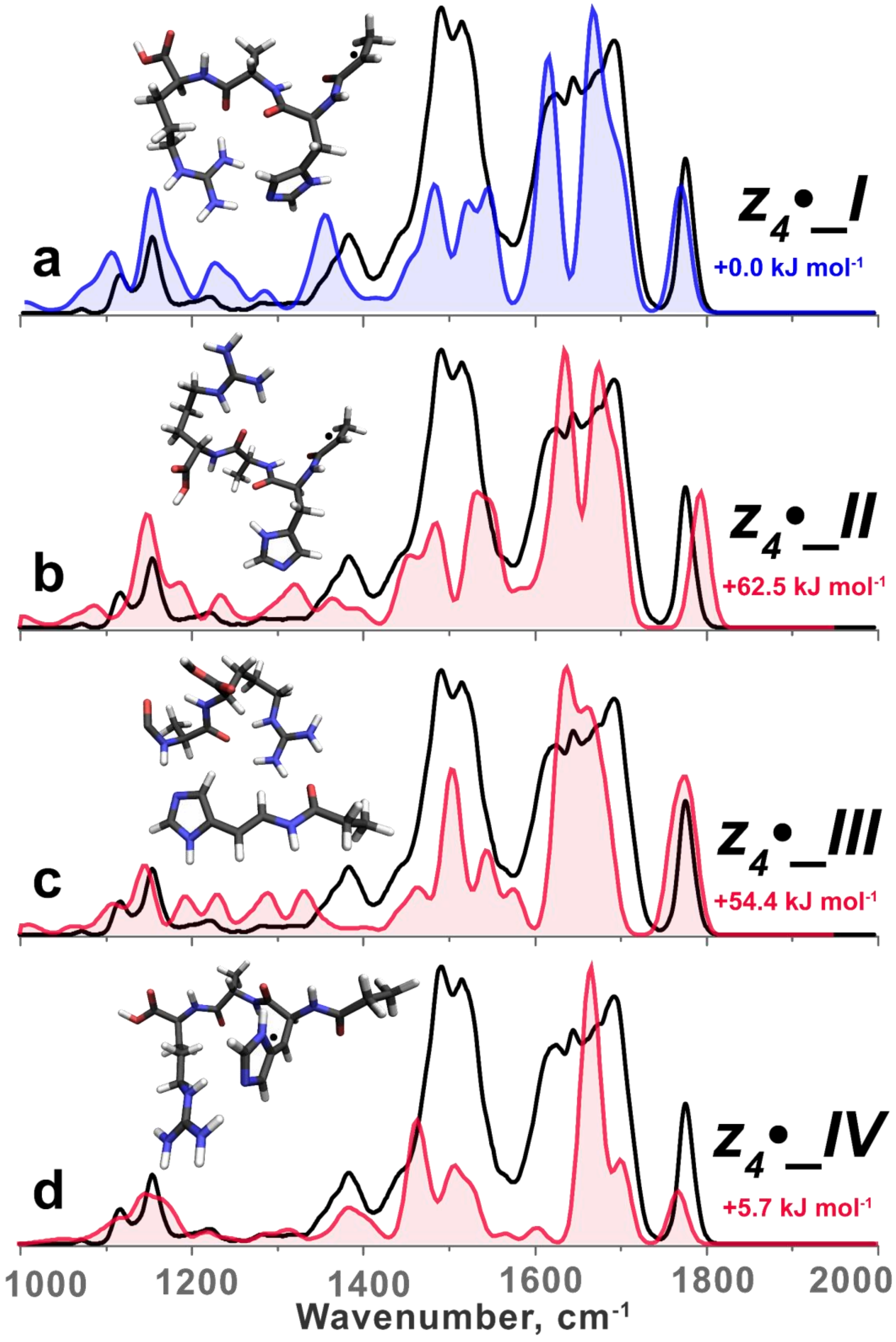


**Supplementary Figure 7 | The infrared spectrum of the $z_4$•$^+$ fragment from ETD of [AAHAR+2H]$^{2+}$.** The experimental spectrum is presented in black in all panels. **(a)** The calculated spectrum of the assigned structure is shown in blue. **(b)**, **(c)** and **(d)** present calculated spectra for structures disregarded on the basis of spectral mismatch. $z_4$•_II is based on a previously proposed[1] structure reoptimized at the level of theory employed in the present study.Calculated structures and relative free energies (298K) are inlayed for each plot.

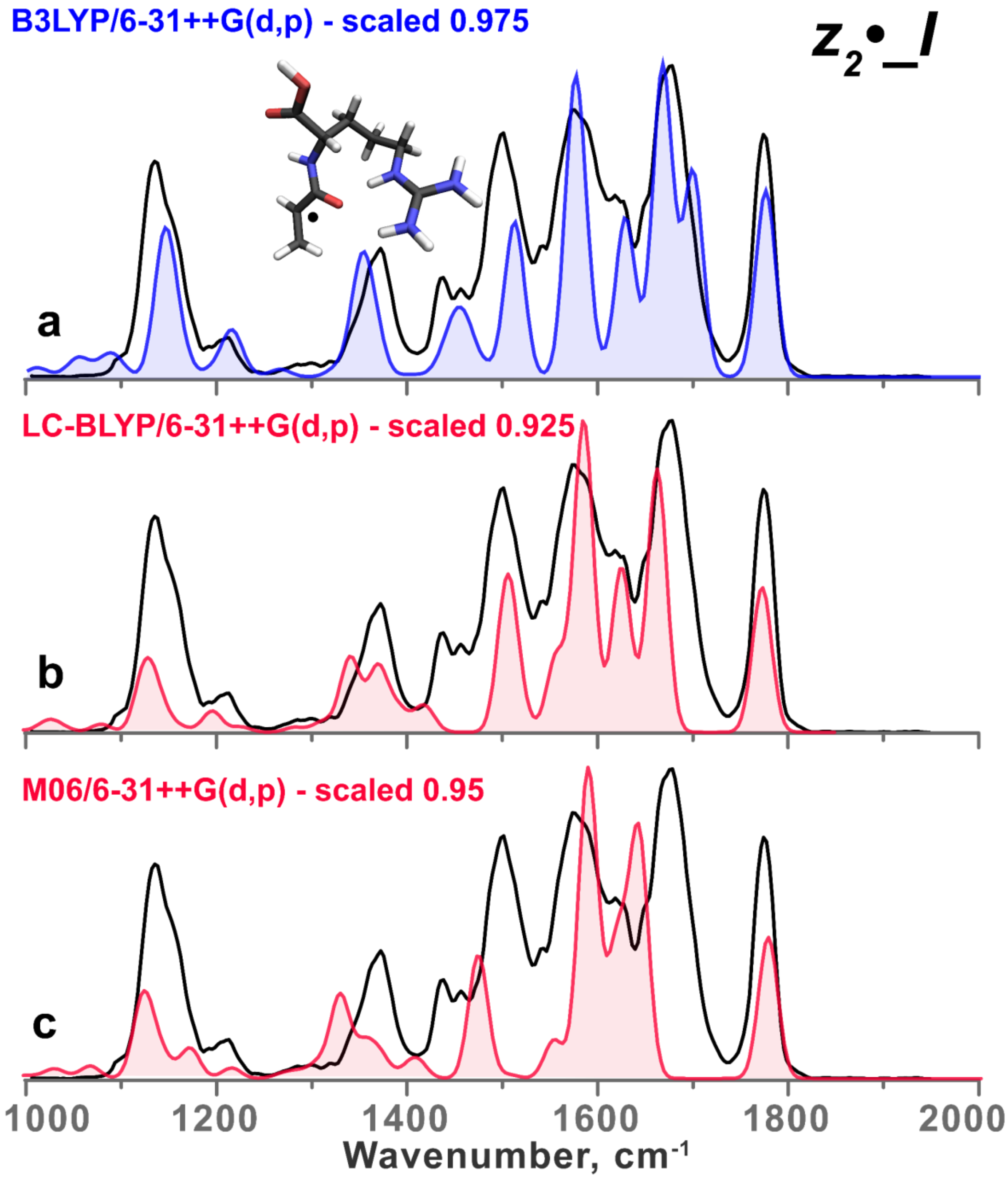


**Supplementary Figure 8 | Comparison of calculated IR spectra for the $z_2$•_I structure using different density functionals.** The experimental infrared spectrum of the $z_2$•$^+$ fragment from ETD of $[AAHAR+2H]^{2+}$ is presented in black in all panels. **(a)** The calculated spectrum for structure $z_2$•_I using the B3LYP/6-31++G(d,p) level. This is the level primarily employed in this study and is shown here to provide the best reproduction of the experimental results. **(b)** The calculated spectrum for structure $z_2$•_I using the LC-BLYP/6-31++G(d,p) level. **(c)** The calculated spectrum for structure $z_2$•_I using the M06/6-31++G(d,p) level.

**Rel. ΔG (kJ mol$^{-1}$)**

| $z_1•^+$ | $z_1•$_I | $z_1•$_II | $z_1•$_III | $z_1•$_IV |
|---|---|---|---|---|
| *B3LYP/6-31++G(d,p)* | 5.4 | 0.0 | 10.1 | 14.7 |
| *M06/6-31++G(d,p)* | 0.0 | 1.1 | 45.7 | 22.6 |
| *LC-BLYP/6-31++G(d,p)* | 0.0 | 0.0 | 49.6 | 19.7 |
| *MP2/6-31++G(d,p)* | 0.0 | 5.8 | 39.5 | 16.7 |
| $z_2•^+$ | $z_2•$_I | $z_2•$_II | $z_2•$_III | |
| *B3LYP/6-31++G(d,p)* | 0.0 | 10.2 | 26.5 | |
| *M06/6-31++G(d,p)* | 0.0 | 11.8 | 26.7 | |
| *LC-BLYP/6-31++G(d,p)* | 0.0 | 10.8 | 25.5 | |

**Supplementary Table 1 | Comparison of calculated relative free energies using different density functionals.** Relative free energies (298 K) for selected calculated structures of the $z_1•^+$ and $z_2•^+$ fragments from ETD of $[AAHAR+2H]^{2+}$.

## Supplementary Note 1 | ETD and IRMPD Product Ions.

IR photodissociation of the doubly protonated precursor ion $[AAHAR+2H]^{2+}$ (m/z 263) produced mainly b- and y-type ions, including doubly protonated $y_3^{2+}$ and $y_4^{2+}$ ions. The $z_3•^+$ and $z_4•^+$ fragments resulting from ETD of $[AAHAR+2H^+]^{2+}$, upon IRMPD, produced primarily the $z_2•^+$ ion. The ETD-generated $z_2•^+$ ion produced an ion corresponding to neutral $CO_2$ loss and ions at m/z 100 and 87. Lastly, the $z_1•^+$ fragment resulting from the ETD reaction mostly produced an ion at m/z 87.

## Supplementary References


1. Ledvina A, Chung T, Hui R, Coon J, Tureček F. Cascade Dissociations of Peptide Cation-Radicals. Part 2. Infrared Multiphoton Dissociation and Mechanistic Studies of z-Ions from Pentapeptides. *J Am Soc Mass Spectrom* **23**, 1351-1363 (2012).

2. Leavitt CM, DeBlase AF, Johnson CJ, van Stipdonk M, McCoy AB, Johnson MA. Hiding in Plain Sight: Unmasking the Diffuse Spectral Signatures of the Protonated N-Terminus in Isolated Dipeptides Cooled in a Cryogenic Ion Trap. *The Journal of Physical Chemistry Letters* **4**, 3450-3457 (2013).
