# Supplementary material for: Structural identification of electron transfer dissociation products in mass spectrometry using infrared ion spectroscopy
Source: Nat Commun. 2016 Jun 9;7:11754. doi: 10.1038/ncomms11754 (PMC4906228; doi:10.1038/ncomms11754)
Supplement: Supplementary Data 1 — Optimized coordinates for assigned structure z1 [file ncomms11754-s2.docx]

**Optimized coordinates of z_1_•_I**

C -2.29185400 0.77109200 0.27385400

C -1.13985700 1.43001900 0.95508100

C -2.30952900 -0.65331600 -0.00048900

H -1.52397100 2.24590800 1.57679900

H -0.65533800 0.71269500 1.62665000

C -0.05906300 2.04377600 0.01690300

O -1.33940700 -1.41319500 0.14410300

O -3.49484600 -1.09578700 -0.44678600

H -0.50041800 2.83968100 -0.59313300

H 0.69650100 2.51537000 0.65638500

C 0.60617600 1.04245400 -0.94099100

H -0.11275400 0.70257200 -1.69288400

H 1.42264400 1.51568700 -1.49319100

N 1.09839500 -0.16878700 -0.26972600

H 0.34477400 -0.81842400 -0.00053300

C 2.35266900 -0.49586500 0.00625100

N 3.36890300 0.37629500 -0.12720000

N 2.63362800 -1.74498800 0.42693600

H 3.20408300 1.35267600 -0.31333800

H 4.32688200 0.05937700 -0.13325100

H 3.50014300 -1.95507300 0.89951100

H 1.91570800 -2.45532200 0.43644600

H -3.16768600 1.34395300 -0.01559800

H -3.43428500 -2.05384400 -0.60613400
