# Supplementary material for: Structural identification of electron transfer dissociation products in mass spectrometry using infrared ion spectroscopy
Source: Nat Commun. 2016 Jun 9;7:11754. doi: 10.1038/ncomms11754 (PMC4906228; doi:10.1038/ncomms11754)
Supplement: Supplementary Data 2 — Optimized coordinates for assigned structure z2 [file ncomms11754-s3.docx]

**Optimized coordinates of z_2_•_I**

C -1.90854200 2.74493600 0.10615900

C -1.08054500 1.56655900 -0.05630400

C -3.33306300 2.74402900 0.54137400

O 0.11393200 1.66826700 -0.44940400

N -1.62436600 0.34954300 0.24043000

C -0.98821000 -0.92168400 -0.09186200

C -1.94262200 -1.86020700 -0.83581500

O -1.56206400 -2.80711300 -1.48125200

O -3.24454900 -1.54606600 -0.65005200

C -0.43196000 -1.68690600 1.14182900

C 0.70863200 -1.01932100 1.93215700

C 2.09165200 -0.99238100 1.25976400

N 2.15305200 0.05110900 0.23181200

C 3.22708200 0.42553900 -0.44817500

N 3.14207400 1.50684200 -1.24724100

N 4.39446400 -0.23663400 -0.34789500

H -1.40402100 3.67697700 -0.12471600

H -3.45707500 2.31431400 1.54688100

H -3.73343700 3.75873600 0.56873000

H -3.96933400 2.15591300 -0.13671300

H -2.60321200 0.31166500 0.49410700

H -0.18225200 -0.71242900 -0.79817700

H -3.78806800 -2.20292600 -1.12077800

H -0.10542400 -2.67295600 0.79301800

H -1.26597300 -1.85791100 1.83255800

H 0.43275000 -0.00247000 2.23680400

H 0.82701500 -1.58701500 2.86096000

H 2.30562200 -1.97399100 0.81451100

H 2.85348000 -0.79056000 2.02344400

H 2.25479100 1.99126400 -1.32465300

H 3.85598600 1.71439000 -1.92826600

H 4.47438000 -1.06987900 0.21316900

H 5.23371400 0.10791700 -0.78828400

H 1.30138500 0.61886200 0.04856500
