# Supplementary material for: Structural identification of electron transfer dissociation products in mass spectrometry using infrared ion spectroscopy
Source: Nat Commun. 2016 Jun 9;7:11754. doi: 10.1038/ncomms11754 (PMC4906228; doi:10.1038/ncomms11754)
Supplement: Supplementary Data 3 — Optimized coordinates for assigned structure z3 [file ncomms11754-s4.docx]

**Optimized coordinates of z_3_•_I**

C 0.43771300 -3.57274100 -0.81850400

H 0.58063400 -3.73533200 -1.88360600

C -0.78301500 -4.13440100 -0.14487600

C 1.40887900 -2.77842200 -0.07334400

H -1.35036600 -4.73723600 -0.85763700

H -0.48018400 -4.80424700 0.67250400

C -1.68689200 -3.06187400 0.40895300

O 1.31307400 -2.59289200 1.15739200

N -1.40393200 -2.39871200 1.58471900

C -2.84870700 -2.48669700 -0.05427100

H -0.50426700 -2.49149300 2.05454800

C -2.35960600 -1.46298100 1.78662600

N -3.26045100 -1.48361400 0.80832000

H -3.41438100 -2.74123800 -0.93936000

H -2.37180000 -0.80461200 2.64378300

N 2.42876100 -2.23745000 -0.80966000

H 2.40114600 -2.32260700 -1.81831900

C 3.33955100 -1.23055200 -0.27113900

H 3.26354300 -1.30394500 0.81571300

C 4.78015100 -1.48452300 -0.72698400

C 2.85593100 0.15089100 -0.76201900

H 5.45310600 -0.73473900 -0.29990800

H 5.10716400 -2.47327900 -0.39514400

H 4.85686300 -1.42528400 -1.81647200

O 2.80063500 0.41263100 -1.96310300

N 2.47776900 1.02942700 0.20393700

H 2.56877400 0.78824900 1.18472500

C 2.11724200 2.40977300 -0.08923600

H 2.90388700 2.86622500 -0.70368700

C 0.79082200 2.56897400 -0.87819100

C 2.08299700 3.15630000 1.24037300

H 0.61112000 3.64281800 -0.99980400

H 0.96217100 2.14667800 -1.87221000

C -0.41874400 1.88840800 -0.22474200

O 1.89792900 4.47775100 1.06677400

O 2.19876300 2.63127100 2.32817100

H -0.24389500 0.80611900 -0.18941700

H -0.55249700 2.22400600 0.81128400

C -1.71028400 2.16582500 -1.00438600

H -1.98568900 3.21977200 -0.88382100

H -1.55823900 1.97155000 -2.07447800

N -2.79652200 1.32241200 -0.49152000

H -2.54577500 0.42464000 -0.08362100

C -4.11244200 1.53404700 -0.60276300

N -4.58273000 2.63128500 -1.22665600

N -4.95157300 0.64522300 -0.06302600

H -5.57169700 2.82408600 -1.26263000

H -3.96735300 3.27586100 -1.69583400

H -4.54875800 -0.21214400 0.36854300

H -5.94875500 0.73063100 -0.18704800

H 1.91120100 4.90327200 1.94223800
