# Supplementary material for: Structural identification of electron transfer dissociation products in mass spectrometry using infrared ion spectroscopy
Source: Nat Commun. 2016 Jun 9;7:11754. doi: 10.1038/ncomms11754 (PMC4906228; doi:10.1038/ncomms11754)
Supplement: Supplementary Data 4 — Optimized coordinates for assigned structure z4 [file ncomms11754-s5.docx]

**Optimized coordinates of z_4_•_I**

N -2.25178800 -1.94300800 -0.33139500

H -2.09635800 -2.79776000 -0.85588000

C -3.64617800 -1.66005600 -0.01855900

H -3.68445000 -1.21161400 0.97893600

C -4.32936500 -0.72489800 -1.04996900

C -4.38447100 -2.99728800 0.03206500

H -4.25218800 -1.19441800 -2.03711700

H -5.39673300 -0.66782300 -0.80758600

C -3.72770200 0.68580300 -1.12398100

O -5.60506700 -2.87139300 0.58082100

O -3.93578900 -4.03991600 -0.39439500

H -2.64175100 0.62287700 -1.25344400

H -4.12074300 1.17927300 -2.02112600

C -4.06186900 1.55803700 0.09782400

H -5.14509000 1.67631800 0.19956100

H -3.70640300 1.10761900 1.02748000

N -3.49265000 2.90736900 0.00227500

H -3.98425800 3.57109800 -0.57971000

C -2.26247600 3.26619600 0.41457300

N -1.46916900 2.40194200 1.05003000

N -1.83009400 4.52466700 0.23372100

H -0.55430700 2.73559700 1.32256300

H -1.58374400 1.38492500 1.01774300

H -0.81154600 4.69906400 0.28608600

H -2.42815600 5.21737600 -0.18962700

C -1.20659900 -1.37964300 0.32332700

C 0.13089300 -2.11741700 0.12125200

O -1.33587800 -0.40250700 1.06983200

N 1.28377300 -1.25568200 0.31262600

H 0.14271900 -2.49383000 -0.91043000

C 0.21289100 -3.29912900 1.10480900

H 1.99190600 -1.54181500 0.98845200

H 0.18912500 -2.94215500 2.13900900

H -0.62111600 -3.98981700 0.95830400

H 1.14375200 -3.85107300 0.94790100

C 1.66051400 -0.33126100 -0.60699500

C 3.09011500 0.23296100 -0.37049300

O 0.96339800 0.00045600 -1.56573000

N 4.08687100 -0.74669600 -0.79068400

H 3.23147900 0.36519100 0.70719200

C 3.31205200 1.57153400 -1.10251400

H 4.39008100 -0.74363000 -1.75556100

H 4.38764600 1.78133000 -1.11220700

H 2.98077600 1.47041500 -2.14029900

C 2.57148400 2.70850300 -0.47151700

N 3.08323400 3.47905100 0.56277400

C 1.29877700 3.18693300 -0.65818100

H 4.01588700 3.41629400 0.94793000

C 2.12544300 4.36399300 0.95102500

N 1.02478500 4.20904900 0.23373300

H 0.57079400 2.83332900 -1.37301500

H 2.27616100 5.08941500 1.73758900

C 4.49898000 -1.76665100 0.04569700

C 5.53159500 -2.64545600 -0.47337100

O 3.99997200 -1.89288200 1.18103800

H 5.91162300 -2.46802200 -1.47758700

C 6.08280800 -3.76728600 0.32469900

H 5.92436200 -4.72815100 -0.18588400

H 7.17086700 -3.66540100 0.44229300

H 5.62091000 -3.80848600 1.31276600

H -6.04043100 -3.74228800 0.56341000
